# Book Reviews

**Published:** 1821-12

**Authors:** 


### CRITICAL ANALYSES
### OF
### RECENT PUBLICATIONS, IN THE DIFFERENT BRANCHES OF MEDICINE AND SURGERY.

"I would have men know, that, though I reprehend the easie passing over of the causes of things "by ascribing them to secret and hidden vertues and properties; (for this hath arrested and laid "asleepe all true enquiry and indications;) yet I doe not understand but that, in the practical "part of knowledge, much will be left to experience and probation, whereunto indication cannot "so fully reach: and this not only in *specie*, but in *individuo*. Yet it was well said, *Vere scire* "*esse per causas scire*."—BACON.

*Essays on Hypochondriasis, and other Nervous Affections.* By JOHN REID, M.D. Member of the Royal College of Physicians, London; and late Physician to the Finsbury Dispensary. *The Second Edition, with considerable Additions.* 8vo. pp. 440. Longman and Co. London. 1821.

IF hypochondriasis be, in reality, what many people pretend it to be—a disease of the mind,—we have no hesitation in saying, at once, that the best cure for it will be found in Dr. Reid's book. We have ourselves suffered cruelly from the blue devils, and know full well how to appreciate the man who can make us laugh under circumstances so distressing. But if, in addition to giving us such a blessing, the same individual can successfully combat our fears, and quiet our apprehensions: if he can turn our grins into smiles, and our hatred to mankind into a longing for society, then he has worked a miracle indeed, and the cure is certain. Unfortunately, however, the *metaphysics* of medicine in nervous or hypochondriacal affections, are not the only engine on which we can rely for complete success in their treatment. "Vapours" may be only a figurative expression; and good folks in old days may have fancied that the disease, which that name was intended to designate, is equally figurative; but, in order to produce "the vapours" of old, or the "blue devils" of more modern times, there must be a derangement somewhere in the economy of our animal system,—let it be stomach, liver, spleen, or any other organ;—and a derangement of this nature is not to be cheated, as it were, into a "righteousness of action" by any well-written essay, however humorous or replete with frolics. We must have the blue-pill and calomel, and purgatives, and tonic draughts, and diet, and alterative powders, and . . . . we dare not proceed; but unquestionably we must have a something out of the druggist's shop, besides one or two leaves out of Dr. Reid's book. We shall then be sure of affording to our splenetic patients the best chance of recovery which human skill can afford. A late and very learned lord, at the conclusion of a consultation held on his wife by some eminent physicians, thus apostrophized her ladyship :—" So, so, my lady! your complaint is nothing but the nerves, after all! You have got the nerves! A pretty complaint, truly! Now, let me tell your ladyship that I will not suffer any one of my family to be in the fashion ; so I advise your ladyship to get rid of your nerves as soon as possible, or d— me if I don't get rid of you." One of the physicians who had lingered somewhat behind the others, overhearing this unexpected harangue, and trembling for the fate of his patient, thrust his head between the half-closed foldings of the door, and exclaimed, " Softly, my lord! *nerves* form a part of our body, just as much as flesh and blood ; and, when we say that they are affected, it follows that means must be taken to relieve them, exactly as your lordship has recourse to the bottle to prevent the blood from being impoverished, or to rich viands to preserve the rotundity of your stomach."

Thus much we would say to Dr. Reid, had we not already analyzed his work, on the appearance of the first edition of it in 1816. We then gave a full account of its contents; and we can only limit ourselves, on the present occasion, to a few desultory observations on the additional matter contained in the second edition.

The first essay, *on the Influence of the Mind,* is copied verbatim from the first edition.

In the next essay, *on the Power of Volition,* besides some short remarks on madness, the effect of the enfeebled power of volition in an actual disease of the mind, and the importance of cheerfulness and hilarity, we find the following curious note:

" Of a late member of the English bar, whose strong original powers of mind had been obscured and enfeebled by the sensuality of his habits, it has been related, that, in the extremity of his last illness, when the shadows of death were fast coming over him, he, with a blasphemous audacity, swore, by God, that he would not die! In this state of morbid and impious rage, he struggled out of his bed, tottered down the stairs, and fell lifeless in the passage. From the exclamation of this unfortunate man, it would seem as if he fancied that he held the reins of life in his hands, and could arrest at will the rapidity of its descending career.

" It is remarkable that a similar story is related in Spence's Anecdotes, recently published, of Salvini, ' an odd sort of man, subject to gross absences, and a very great sloven. His behaviour in his last hour was as odd as any of his behaviour in all his life-time before could have been. Just as he was departing, he cried out in a great passion, ' Je ne veux pas mourir, absolument.' "

*On the Fear of Death,* the subject of the third essay, Dr. Reid has greatly enlarged his former observations. It is a curious circumstance that those persons should be, in general, found to dread most their departure from this world, to whom it has proved less productive of enjoyment: nor is it less singular that, the more serious and aggravated are our sufferings, generally speaking, the more strongly we cling to life. The apprehension of dying, however, which forms so prominent a symptom of hypochondriasis, is, in most cases, in the inverse ratio of the danger attending that disease. Dr. Reid quotes an example in illustration of this morbid sensibility, which may be assumed as a good specimen of most of the common cases of hypochondriasis.

"At the present moment I know a person of this class, whose conversation exhibits a general superiority of mind, attended however with a partial imbecility. His good sense deserts him only upon the subject of his health. His own opinion of his disease constitutes the worst part of it. His complaint appears to be seated in the stomach, and apprehension seems to be in great measure the creature of indigestion. There is no circumstance attending his ailments that indicates danger, or is inconsistent with a fair chance of longevity ; and yet, for a considerable period, he has been decidedly of opinion, without being able to give any reason for the inflexible belief, that he shall never recover, and has been long, in his own imagination, trembling on the very edge of the grave."

Our author, we think, has also happily described, in his peculiar style, by which we are reminded of the forensic eloquence in a sister island, the state of perverted intellectual vision in an hypochondriac.

"Instruments have been invented by which the most remote objects of vision may be drawn so near to the eye, as to seem almost in contact with it. Something analogous to this power exists in the mental mechanism of many an hypochondriac, by means of which he approximates to himself events at the greatest distance either in prospect or in retrospect, either before or behind him in the road of life. This power contracts the interval of time, as a telescope does that of space. The most remote calamity which he anticipates, he feels as if it were actually crushing him with its weight. From being in the habit of contemplating, with a morbid intensity, the close of his earthly career, he forestals almost every day of his life the agonies of dissolution. The spectre of human mortality is continually presenting itself before him in the full dimension of its horrors, so that it is no wonder if actual death be often occasioned by the appalling apparition. It is similar with regard to the past: although the substance of some great calamity has long gone by, its lingering shadow still continues to darken his path. Years make no impression upon the immutability of his feelings. The ideas of recollection are, in general, less lively than those which are produced by an immediate operation upon the senses. But with a certain class of hypochondriacs it is quite otherwise. The pictures drawn upon the fancy exhibit a more distinct and vivid colouring than belongs to the realities of life."

But it is not the fear of death alone which is apt to overshadow the mind of the hypochondriac. He is often the slave of fear which has no specific object. He trembles under the weight of indefinite apprehensions. He has no resolution, no enterprise. He is imprudently cautious; and the foresight of possible evil shuts him out from the chance of probable advantage.

Of the two succeeding essays we shall say nothing. Their morality will recommend them to the attention of the well-disposed; but, although evidently intended to point to physical objects, no medical man will derive much useful information from them for the purpose of practice.

Of the sixth essay, which treats on *Solitude*, we confess to have scarcely collected the meaning. Indeed, Dr. Reid, writing under the disadvantage of a thorough conviction that the readers of a Magazine, in which his essays originally appeared, demanded something more than the plain unmeaning technicalities of his brethren, has not unfrequently adopted a mystical and metaphorical diction, which may perchance subject him to the derision of many. The article on solitude concludes thus: —" There is an *antiseptic* power in an active benevolence, which counteracts the putrescency of melancholy, and has in some instances proved an antidote even to the gangrene of despair ! !"

We dilated so amply in our former review on the subject of the next essay, entitled *Excessive Study*, that we must spare our observations upon it in this place. Dr. Reid relates the anecdote of Carlini, who had consulted an eminent practitioner at Paris with regard to what might be the best remedy for the depression under which he laboured, and who was directed to go and see his own performance at the theatre. We also consulted a French practitioner of great eminence, some years ago, under similar circumstances, though by far more distressing ; and were desired to quaff, frequently in the day, large potations of coffee-water! The practitioner himself is well known as the most hypochondriacal person in France.

Of the two faults which we pointed out on a former occasion in the essay on *Vicissitude*, the author has corrected that which was a venial one,—namely, he has made a *short* chapter longer; but he has omitted to reform the more important fault, relative to the too-frequent use of fantastical allusions and figurative language. Who would ever have compared " the supposed torpor of melancholy, to a child's top after it has been lashed into the most rapid agitation ?" How can " the hours of enjoyment be heard ?" On what metaphysical map shall we find traced the " *equinoctial* condition of the mind ?" It is really not encouraging for us poor reviewers to see our counsels thus neglected.

Dr. Reid has added to the present edition a whole essay on *Sleep*, preceding, as we trust it may always do with our readers and ourselves, the *Want of Sleep*. Against the latter malady, (for *pervigilium* must be considered as such,) the author has no remedy to offer except a clear conscience.

"Some years ago, I was called to one of the most notorious characters in London. He was an hypochondriac, the principal feature of whose complaint was an obstinate watchfulness. He had, in rotation, tried nearly all the doctors, great and small, in the metropolis; but they seemed all to have been equally inefficient. No medicine could be applied to the seat of his disease: no contrivance of art could lull his conscience to repose. With all his dexterity in fraud, for which he was perhaps unrivalled, he was unable to cheat himself. In our public courts of justice he often, by the application of technical subtleties, braved the judge upon the bench; but he trembled before the secret and more formidable tribunal that was established within his own breast. The laws of England may be evaded, but those of nature cannot. Junius says somewhere that, upon his honour, he never knew a rascal that was a happy man. No one, I believe, ever knew a rascal that was habitually a sound sleeper."

We commended the essay on *Intemperance* on a former occasion; and we see nothing to detract from the opinion we then expressed of its merits. The observations on the use of opium will be read with interest. To the examples of persons swallowing daily large quantities of that drug, which the author has brought forward, may be added another which is to be found in the two last Numbers of the London Magazine, where it appears, from the confessions of the individual himself, that a veteran opium-eater had brought himself to take at last sixteen ounces of laudanum in the course of twenty-four hours.

We pass over the next four essays on *Abstinence; Morbid Affections of the Organs of Sense; Physical Malady the occasion of Mental Disorder;* and on the *Atmosphere of London:* in order to come to the more practical part of the work.

The first useful remark we meet with in the sixteenth essay, respecting *Dyspeptic and Hepatic Diseases*, is that which relates to over-indulgence in the pleasures of the table, and to the diseased condition of the respiratory organs, considered by the author to be the result of gluttony.

"In the more self-indulgent classes of society, which, although not in general so denominated, ought, in a rational and moral view, to be regarded as the lower orders, it may be remarked, that coughs originate not so frequently from a diseased condition of the lungs, as from a depraved state of the principal and more immediate organ of digestion. Hence arise those chronic coughs which are almost universal amongst the obstinately intemperate in eating as well as in drinking. Such coughs become particularly troublesome early in the morning, when the tone of the stomach has not been as yet duly excited by the natural and artificial stimulants of the day. The violent paroxysms of coughing often produce a tendency to vomiting. In such cases emetics give relief, which are on that account, by these spendthrifts of constitution, so frequently resorted to, as in some instances to become a part of their habitual regimen. An emetic relieves a person for a time from the filth and burthen of a debauch; but this medicinal mode of purification is scarcely less injurious than intemperance itself. I knew a man whose conduct in every other respect was highly exemplary, who was in the habit of clearing his stomach in this manner, on returning from a dinner-party. The violent action of an emetic under these circumstances was the immediate occasion of his death."

Warm diluents, Dr. Reid asserts, form a less injurious mode of relief from the effects of indigestion. The celebrated Burke was as much in the habit of refreshing *himself*, (the author has it "herself:" does he mean to insinuate that B. was an old woman?) by draughts of hot water, as the no less celebrated Pitt by potations of wine. But even the long application of hot liquids may be supposed to affect the coats of the stomach in the same manner "as the fingers of a washer-woman are *soddened* by a habit of immersion in tepid water."

On the hacknied subject of tea-drinking, we must quote the author's own expressions. His opinion on this subject is an addition to the first edition of his work.

"In spite of these observations, I am still inclined to think that there are many cases in which a taste for tea ought to be encouraged rather than condemned. This taste has a tendency to preclude the more prevalent, and after all more mischievous, propensity for vinous stimulation. Many persons, distinguished for their longevity, have been known to indulge habitually in the use of tea; which we may account for, not from its being in itself a wholesome beverage, but from a fondness for it generally implying a distaste for potations of a much more decidedly pernicious nature. Tea will produce, in some very irritable frames, an artificial state resembling intoxication; but it is a cloudless inebriety. Tea removes the film from an eye that has been obscured by a gross and stupifying intemperance, and tends to improve a susceptibility to the true relish of social and intellectual enjoyment."

The connexion between hypochondriasis and some *real* derangement of the constitution, to which we alluded in our introductory remarks to the present article, is acknowledged by Dr. Reid himself in the present edition.

"Between the diseases which form the particular subject of this essay and hypochondriasis, the connexion is intimate and almost inseparable. The assimilation of the food has an important effect upon the regulation of the mind. A sourness of the temper may often be traced to acidities in the stomach. He who does not digest well, is not likely either to act or to feel aright. A morbid secretion of the liver will give a tinge to a man's character, as well as to his complexion. He, whose disposition to goodness can resist the influence of dyspepsia, and whose career of philanthropy is not liable to be checked by an obstruction in the hepatic organs, may boast of a much deeper and firmer virtue than falls to the ordinary lot of human nature. A disorder in the physical part of our frame produces not so frequently a total obscuration as a twilight of the intellect, an intermediate and equivocal state between entire sanity and decided derangement: the state in which a large proportion of hypochondriacal men and hysterical women may be considered as nosologically placed."

Some few additions, but none of great practical utility, have been made to the chapter on *Palsy*, *Idiocy*, &c. Dr. Reid will excuse us if, after having given so ample an account of Dr. COOKE's excellent work on Nervous Diseases, we decline saying any thing on his own short essay. We, indeed, cannot refrain from expressing our surprise that the author should not have even mentioned Dr. Cooke's name. Yet he might have culled from that gentleman's book enough to deck his own essay in " goodly trappings."

The eighteenth essay is on *the Hereditary Nature of Madness.* The nineteenth is on *Old Age.* The twentieth is on *Lunatic Asylums.* The twenty-first, on the *Tendency to Lunacy, and on Lucid Intervals.* These are reprinted nearly verbatim from the first edition. We should have expected, after a lapse of five years, to have found some additional facts or observations on subjects of so much interest, as the result of greater experience and more extended practice; but in this we have been disappointed.

On the subject of *Bleeding*, which is treated of in the next essay, we are completely at issue with our author. The peculiar doctrines he advanced in the first edition, respecting the impropriety of venesection in fevers, are here repeated in the same strain of language, and without any token of better maturity, such as we had reason to expect from a practitioner who must have since had additional opportunities for observation. Dr. Reid is a decided enemy to venesection, except on a very few occasions; and the alledged reasons for his hostility to the lancet are unsatisfactory.

We are better pleased with his remarks on *Pharmacy*, contained in the twenty-third essay. We would wish, in particular, to inculcate on the mind of every practitioner, the following very judicious observations.

" In cases of convalescence from acute disease, to prolong a medicinal course, for the sake merely of still further strengthening, after the natural desire has returned for wholesome and substantial food, is a practice that appears to me contrary to common sense, although it be not altogether so to ordinary routine. Under such circumstances, 'to throw in the bark,' is, to those who are asking for bread, giving a stone. There is no such thing as a permanently strengthening medicine. It is only what nourishes that gives any durable vigour or support. Medicine, as it is usually administered, interferes with appetite before a meal, and with digestion after it. Drugs, although not in general intoxicating, are at best unnatural stimuli; and, of course, are seldom to be resorted to, except in that state of the constitution in which it cannot be duly excited by the ordinary incentives to vital and healthy action."

We have no doubt that, in the practice of physic, we should have been more successful if there had not been a single article of the class of tonics in the materia medica.

The essay on *Pharmacy* is greatly enlarged in this edition, and contains several very apt remarks, no doubt the fruit of personal observation. In what he has stated of the mismanagement of infants, we cordially go along with him; and, so far from following the example of those who have accused our author of hyperbole and exaggeration in what follows, we wish it may have its due effect in improving a branch of medical practice, of which it is not too much to say, that it has hitherto been unaccountably neglected.

"In no department of our profession does the practice of it appear so cruelly absurd as in the mismanagement of infants. I once ventured to observe, that, 'of the cases of mortality in the earlier months of our existence, no small proportion consists of those who have sunk under the oppression of pharmaceutical filth. More infantile subjects in this metropolis are perhaps diurnally destroyed by the mortar and pestle, than, in the ancient Bethlehem, fell victims in one day to the Herodian massacre.' I plead guilty to the charge of rashness and hyperbole, which were brought against this remark when first published, but I wish that the years of experience and reflection, which have since intervened, had convinced me that the remark was altogether destitute of foundation. When we contemplate a church-yard, the earth of which is composed in great measure of the bodies of infants, it is natural for us to fancy, but surely it is not reasonable for us to believe, that those beings were born for no other purpose than to die; or that it is within the design of nature that the pangs of production on the part of the mother should, on that of her offspring, be almost immediately succeeded by the struggle of dissolution. Fault must exist somewhere: it cannot be in the providence of God; it must therefore attach to the improvidence and indiscretion of man. Consequences as fatal originate from ignorance as from crime. Infanticide, when perpetrated under the impulse of maternal desperation, or in the agony of anticipated disgrace, is a subject of astonishment and horror; but, if a helpless victim be *drugged* to death, or poisoned by the forced ingurgitation of nauseous and essentially noxious potions, we lament the result merely, without thinking about the means which inevitably led to its occurrence. Conscience feels little concern in cases of medicinal murder. The too ordinary habit of jesting upon these subjects in convivial or familiar conversation, has an unhappy tendency to harden the heart, and inclines us to regard, with an inhuman and indecorous levity, those dark and horrible catastrophes which too frequently arise from professional ignorance or mistake."

On the remaining five essays we shall offer no remark. They are generally written in a more sober style, and contain many useful hints and practical observations. Their subjects are, *Ablution; Bodily Exercise; Occupation; Real Remedies, and a Remedy for those of the Imagination.* We highly approve of the author's opinion respecting the utility of warm bathing.

"The warm bath has a remarkable influence in composing the mind when in that state of violent irritation, which often leads to the use of laudanum or some equally deleterious opiate. This remedy has been for many years considered, at the Retreat at York, as of greater efficacy in certain cases of insanity than all the other medicinal means which have been employed. There is no agent which, equally with the tepid bath, is calculated to promote the general tranquillity of the constitution. It will often induce sleep when the more direct and accustomed opiates fail, and, with all its beneficial tendency, it is followed by none of those evil effects that are apt to arise from the drugs more generally employed to allay uneasiness, to restore composure, and to conquer the obstinacy of an involuntary and unnatural vigilance. The notion that the warm bath is relaxing, may in a great measure be derived from the effect which it is observed to produce upon inanimate matter; as if the nerves and muscles of the human frame were like the strings of a musical instrument. The warm bath is, in many cases, a congenial and salutary cordial; it animates torpor and elevates depression; on which account, when intemperately employed, or in cases where there is already a too vigorous excitement, there is a chance of its proving deleterious. In furious mania, for instance, it has been known to produce mischievous effects."

Our own experience coincides with that of the author in respect to the beneficial effects of the warm bath; by which we mean water at a temperature no less than 97 degrees, and often 99 and 100.

Dr. Reid has added to the present edition of his work an Appendix, containing a few interesting passages from notes and additions made to a German translation of the first edition, by Dr. HAINDORF at ESSEN. It cannot be expected that we should enter on the consideration of these fragments; but we will not deny ourselves the pleasure of quoting one or two passages, from which it will appear evident that the translator had caught the true spirit of his author, and, in his own original remarks, endeavoured to think, reason, and write like him.

On the subject of insulted pride as the source of mental disease, we have the following anecdote:

"It happened, at W——, that a gentleman, whose pride was well known, was one evening refused admittance to the nobility's box. He was highly incensed; and the next morning it was found necessary to put him into an asylum for lunatics. No art could there succeed in restoring the disturbed equilibrium of his mental powers; and, after a residence of ten years, he died there in a state of misery and stupefaction."

Nor were we less amused at the ready manner in which the German Professor cured a young lady of a disorder, the removal of which would, but for his interference, have been attributed to the miraculous power of animal magnetism.

"In the town of W——, I was one evening called to visit a young lady, who was supposed to be dangerously ill with convulsions. Her relations told me that, other remedies having failed, she had for some time past been magnetised by her medical attendant. I found her lying on the sofa in a kind of extasy, and was informed that this state recurred several times in the day, but not with so much violence as then. She was accustomed, during the fit, to speak of higher things, and appeared quite unconscious of the world in which she lived. All belonging to her were assembled about her, to contemplate the wonder, as if she were a saint. As the lady had, in the paroxysms of a disease which was supposed to deprive her of her senses, placed herself in the most elegant attitude on the sofa, had arranged her fine light hair very nicely, and had bestowed great attention on the choice of her attire altogether, — these circumstances, added to the state of her pulse, inspired me with some doubts respecting her unconsciousness and supernatural visions. I therefore endeavoured to comfort those about her, by saying, 'Be under no uneasiness: this is an ordinary case. Magnetism is not necessary to cure it. I will only cut off the patient's hair, and apply a cold cataplasm.' The instant I drew out my case of instruments, the lady roused herself from her ecstasy, and has never had a similar attack. She took some strengthening medicine, and is now a stout and healthy young woman."

---

*A Practical Treatise on the Inflammatory, Organic, and Sympathetic Diseases of the Heart; also on Malformations of the Heart, Aneurism of the Aorta, Pulsation in Epigastrio, &c. &c.* By HENRY REEDER, M.D. Member of the Medical and Chirurgical Society of London, and extraordinary Member of the Royal Medical Society of Edinburgh. 8vo. pp. 276. Causton, London, 1821.

WE would ask Dr. Reeder whether, at his age, he has actually seen a sufficient number of cases of diseases of the heart as to enable him, either to confirm what other writers have advanced, or to bring forward new facts and more correct views respecting that class of organic affections. If he was not qualified to do either, wherefore has he undertaken the task of writing a *practical* treatise on the subject? That a veteran practitioner like Corvisart, who had placed himself at the head of a clinical establishment, wherein scarcely any other case of disease than those of the heart were admitted for treatment, should, after a long series of years of experience, write upon them, we can readily comprehend; and we rejoice at the fact. That an anatomist of the tried skill of Pelletan should indite essays on the same class of morbid affections, no one will wonder at, since the opportunities of seeing and watching the progress of these affections, which he enjoyed in a large hospital for many years, are known to have been numerous. That Dr. Warren, of Boston in America, should venture on some original remarks respecting organic diseases of the heart, after having seen and recorded several cases of them, most industriously collected and accurately described, even Dr. Reeder will admit to be a proper and a fortunate circumstance. That Laennec, than whom few persons since the days of Morgagni have more diligently studied the morbid structure of the thoracic viscera, should feel anxious to give to the world the result of his investigations, one can easily understand. But that a medical man, who professes to have nothing either very new or very singular to impart to us, on the subject of these very diseases, should set himself down to compose a treatise upon them, we confess to be unable to comprehend. Does Dr. Reeder think that neither Burns's book, nor the translation of Corvisart's work, are a sufficient guide for the English reader in the study and treatment of diseases of the heart? If so, we must next inquire into the nature of the substitute he has himself brought forward for these two works; and examine into the merits of a performance with which Dr. Reeder has probably intended to supply the deficiencies left by the above illustrious writers.

Dr. Reeder's Treatise on Diseases of the Heart presents a remarkable feature. It contains no classification of those maladies. The book is simply divided into so many essays, of which there are twelve, and each of which might stand of itself; for it has no connexion with that which precedes it, nor does it portend what is to follow.

The principal subjects of these twelve essays may be thus enumerated:—1. Carditis. 2. Angina Pectoris. 3. On Change in Structure of the Valves of the Heart and large Arteries. 4. Enlargement of the Heart. 5. Diminution in the Size of the Heart. 6. Adhesion of the Pericardium to the Heart. 7. Polypi in the Cavities of the Heart. 8. Sympathetic Affections of the Heart. 9. Malconformations of the Heart. 10. Hydrops Pericardii. 11. Aneurism of the Thoracic Portion of the Aorta. 12. Pulsation in Epigastrio.

I. *Carditis.*—The heart, like most other parts of our fabric, is liable to inflammation. Carditis will manifest itself suddenly at times; and occasionally it will be found to have made its approach insidiously. From the consideration of the intensity of the symptoms attending it, Dr. Reeder is disposed to divide carditis into the " acute variety" and the " sub-acute form." Of each of these he has detailed the symptoms. Those of the latter are only different in intensity from the characteristic symptoms of the acute variety, which are described by our author in the following extract:

" *Symptoms of the most Acute Variety.*—Those which usually demonstrate the existence of the most acute form are—general pyrexia; extreme anguish or pain, with a sensation of heat or burning, in the region of the heart, accompanied with a labouring, and sometimes a jarring, sensation in its action. Most commonly there is violent palpitation of the heart, yet on some occasions it is, in a great measure, absent; and syncope, more or less complete, takes place; or one of these not infrequently alternates with the other. The pulse is rapid, hard, and often irregular and intermittent; great anxiety and restlessness or jactitation are also present; and the patient's countenance has an expression indicating the greatest distress. Sometimes there is an entire inability to lie in the horizontal posture, and the person then experiences some slight alleviation of his sufferings by leaning forward; and, in other cases again, no such uneasiness is produced by that position. Vomiting, too, on some occasions, takes place; and delirium not infrequently supervenes. Wandering pains have, in some instances, been felt in different parts of the body. The respiration, moreover, is accelerated or hurried, by reason of the more rapid transmission of blood through the lungs, and partly also in consequence of the distress experienced in the chest; it is not, however, actually difficult; nor is the pain in the thorax augmented by taking a full inspiration, nor by any slight cough that may sometimes attend."

The sub-acute will often merge into the chronic variety, which is acknowledged to be of very difficult detection. Carditis is often complicated with other inflammatory diseases, particularly of the pericardium or of the lungs. Indeed, Corvisart has asserted that acute carditis never occurs without complication. As for the means of clearly determining the particular organ which shares the diseased action of the heart, we are apprehensive that Dr. Reeder has rather laid down conjectural sources of diagnosis, than positive distinctions warranted by experience.

" When the pericardium is inflamed, and the heart, at the same time, is affected in a similar manner, there are no particular symptoms manifested which enable us to decide whether the former be in reality so diseased."—" It very rarely happens that the pericardium is inflamed alone, and without the heart at the same time being so diseased; yet, if such should occur, there will be pain in the region of the heart, but unattended with the violent palpitation, syncope, and same degree of anguish, which take place in acute carditis."—"When the mediastinum is inflamed, there is pain, with a sense of weight, referred to the middle portion of the sternum, extending downwards to its ensiform cartilage, and accompanied with great anxiety."—"If the diaphragm become inflamed, there will be the superaddition of cough and painful constriction about the præcordia, or a sensation as if a cord were tightly encircled about the lower part of the thorax; the breathing is small, quick, and somewhat laborious, being chiefly performed by the muscles which elevate and depress the ribs, as any muscular action of the diaphragm itself would, under these circumstances, augment the pain very considerably.—When pneumonia, again, is present, there is, besides the symptoms peculiar to carditis, difficulty of breathing, troublesome cough, and perhaps pain in some other part of the chest; and either it, or the pain in the vicinity of the heart, is much aggravated by taking a full inspiration, or by coughing: the sputa at first is mucous or frothy, but afterwards purulent, and sometimes bloody."

We recollect that, when we were examined before the Royal College of Physicians, one of the censors particularly insisted on the distinguishing symptom of inflammation of the heart being *syncope*, which he contended is never observed in cases of inflammation of any other of the thoracic viscera.

Dr. Reeder observes, that, when inflammation affects the heart, it may sometimes spread along the veins and arteries, and give rise to violent palpitations. He should have said, "and produce arteritis," &c.

Of the causes of carditis, we know nothing. The prognosis is always unfavourable. The morbid appearances of the organ affected in this disease are shortly described by Dr. Reeder; but they have been much better recorded by other authors, and it does not appear that Dr. Reeder has derived his information from actual dissection: at least he does not tell us so.

There are some apt observations on rheumatism of the heart, and inflammation of the veins and arteries, in this essay or chapter, which merit consideration. Indeed, we may take this opportunity to observe, that our readers will not be wholly disappointed in regard to this book, in many parts of which we have found some useful information.

*Treatment of Carditis.*—"The acute form of inflammation of the heart requires the early and prompt employment of our most powerful antiphlogistic remedies, the chief of which is blood-letting: this must be used as largely and repeatedly as the violence of the disease seems to demand, or until an obvious alleviation of the symptoms be obtained; and it is oftentimes necessary to carry that remedy to as great, or perhaps greater, extent in this than in any other inflammatory complaint whatsoever. A blister must then be applied over the region of the heart, and repeated if deemed requisite. Purgative medicines, especially the saline ones, should then be administered, so as to induce a moderate degree of purgation. The topical abstraction of blood, by cupping or leeches, may occasionally be enjoined, particularly in the more advanced stages. Refrigerant and diaphoretic medicines may likewise be exhibited; and the patient should be kept cool and tranquil. Digitalis, too, from its power of diminishing the action of the heart and arteries, has been recommended as an adjuvant remedy in this affection, and perhaps with some small prospect of advantage; but, as before observed, blood-letting must form our *summum remedium*."

In the sub-acute form of carditis, the same means may be had recourse to, though not quite to the same extent. The question of blood-letting in *chronic* carditis is not yet settled. Cases in which venesection produced effusion, have been reported on the most unquestionable authorities.

When the heart is affected with rheumatism, Dr. Reeder thinks that the same remedies ought to be had recourse to which are recommended in common carditis. The preparations of mercury should likewise be exhibited, so as to affect the system speedily; a blister must be applied over the region of the heart, or a seton inserted; and the patient should be kept cool and quiet, and subjected to the most rigorous diet.

II. *Angina Pectoris.*—The definition given of this complaint by Dr. Reeder is brief. "By it," says he, "is understood an affection in which there is pain or a sensation of anguish, more or less severe, in the region of the heart, and frequently extending across the chest, up to the shoulder, and down the left side, invading by paroxysms, and therefore alternated with intervals of perfect ease."

The causes which produce angina pectoris are divided into four classes:

"1. An ossified, or otherwise diseased, state of the coronary arteries, whereby their calibre becomes much diminished; or an ossified condition of that portion of the aorta whereat these vessels are given off, so as to lessen the diameter of their aortal orifices.

"2. Ossification and enlargement of the valves of the heart, and of those placed at the origin of the aorta and pulmonary artery; also morbid contraction of the different apertures to which they are attached; and enlargement of the heart accompanying these morbid states.

"3. Aneurism and ossification of the thoracic portion of the aorta.

"4. A disordered state of the chylopoïetic organs, more especially of the stomach, producing indigestion."

Spasm will give rise to pain in the heart; and not unfrequently hydrothorax. A large quantity of fat investing the heart has also been alledged to give rise to angina pectoris; and certainly, where that organ is much loaded, and at the same time incited to increased action, a greater or less degree of anxiety, and sometimes a disposition to syncope, may be induced.

Perhaps, the best part of Dr. Reeder's book is his account of the symptoms of angina pectoris, as it arises from any of the supposed causes detailed above. That the symptoms are taken chiefly from Dr. Heberden, Dr. Parry, and other writers, admits of no doubt. Still they are brought together in a very clear and intelligible manner, and will be referred to with advantage by practitioners, particularly by those who are not possessed of Dr. Parry's book, now so long out of print. But it is curious that neither Dr. Parry nor Dr. Reeder, nor indeed any other writer on this subject, with whom we are acquainted, has mentioned the singular and characteristic symptoms of swelling of the throat, painful deglutition, and hoarseness, which attend diseases of the heart considered as angina pectoris. We have recently had under our care two cases of that disease, in which the above symptoms were very prominent.

Of the treatment of angina pectoris, there is but little to be said. When arising from organical derangement, it admits only of palliation; and, when sympathetic only, the disease producing the sympathetic manifestation should be removed. The treatment of the real angina naturally divides itself into that which is required during the paroxysm, and in what should be effected between the paroxysm. We know of no medicine that will relieve the anguishing pain brought on by the paroxysm of angina pectoris, so soon as a few drops of hyoscyanic acid.

Dr. Reeder has given a summary account of most of the cases of angina pectoris on record; and we do not find that he has added to them any that has fallen under his own immediate observation. The well-marked case of John Hunter is cursorily alluded to by the author.

III. *Change of Structure in the Heart or large Arteries.*—Morbid changes in the structure of the heart are of frequent occurrence; though we are by no means disposed to think, with a certain good-natured physician of a large Dispensary, that every heart which we have occasion to inspect on dissection is diseased.

The semilunar valves of the aorta appear to be more subject to organic derangement in their structure. In our last Number, we inserted a case of dissection by Mr. Marley, in which the valves were found ossified. The left auriculo-ventricular aperture is occasionally found very much contracted, by the deposition of calcareous matter; and, in this case, the mitral valves are also more or less affected. The same process of ossification has sometimes been found to extend to the columnæ carneæ, and to the membrane lining the cavities of the heart.

Bichat has stated, that the right auriculo-ventricular and pulmonary-arterial apertures, and the tricuspid valves attached to the former, are never the seat of organic derangement. Dr. Reeder very properly objects to this sweeping assertion, and proves its incorrectness by an allusion, in particular, to a case contained in the Edinburgh Medical Journal, in which the tricuspid valves were found so agglutinated together, that a small aperture was only left for the passage of the blood.

Excrescences, or fungi, have been found sometimes pending from the aortic, pulmonic, and tricuspid valves, which, by obstructing the free current of blood, became a source of the most alarming symptoms.

Dilatation both of the venous and arterial vessels of the heart, is a morbid alteration of structure most frequently met with.

Dr. Reeder has given a long series of symptoms of the above changes of structure in the apparatus of the circulation, which may be read with advantage. He at the same time recapitulates the diagnostic symptoms of each variety of disease, which we shall beg leave to transcribe.

"*Diagnostic Symptoms.*—Those which may be considered as most distinctive of the left side of the heart being the seat of organic disease, are—the presence of hæmoptysis and extreme difficulty of breathing or dyspnœa, together with the other symptoms already enumerated.

"Such, again, as may be indicative of the existence of enlargement of the semilunar valves, or contraction of the orifice, of the aorta, are —the heart palpitating strongly and violently, while the pulse is small, feeble, contracted, and intermittent: there also being two or three pulsations of the heart to one of the radial artery; and with which if there be the hissing or rustling kind of noise in the chest, as of water passing through too narrow an aperture, precisely synchronous with the pulse at the wrist, the inference that such a morbid state exists may be more certain.

"When the hissing noise, moreover, as that of the rushing of water, is heard betwixt each pulsation of the heart; and there is a jarring or thrilling undulation, or somewhat indistinct pulsation, felt in the region of the heart, betwixt each distinct vibration of that organ, the former arising from the contraction of the auricle, the latter from the action of the ventricle; such may indicate that the obstructive cause is situated at the left auriculo-ventricular aperture: the pulse, at the same time, being small, feeble, and intermittent, or affected in a similar manner to that described in the preceding diagnosis; the other symptoms also attending.

"When the organic lesion, giving rise to obstruction, is existent on the right side of the heart, the dyspnœa is not by any means so considerable as when it is resident on the left side of that organ; neither is there any hemorrhage from the lungs. When the right side, moreover, is the part affected, there may be a more obvious fullness of the venous system and lividity of the external surface, a greater degree of pulsation of the jugular veins and in the epigastric region, and a somewhat greater disposition to enlargement of the liver, than when the left side is diseased; though it is necessary to recollect that such may sometimes be considerable in the latter.

"Sometimes both sides of the heart are diseased at the same time, and then, of course, any particular diagnosis is quite out of the question.

"When the valves on the left side of the heart are ruptured, corrugated, or reticulated, and so permit the regurgitation of blood, extreme difficulty of breathing, and sometimes hæmoptysis, will be present, together with the other symptoms formerly described; but, when the valves on the right side are so affected, no hæmoptysis, and only a comparatively slight degree of dyspnœa, take place. It is very difficult, however, to determine whether the valves are thus affected, or ossified and enlarged, by reason of the great similarity of symptoms produced by both states. Nevertheless, where they are ruptured, shrivelled, or reticulated, there is not two or three pulsations of the heart to only one at the wrist; neither is there, in general, that jarring or thrilling sensation communicated to the hand when placed over the situation of the heart."

The other subdivisions under this head relate to the rupture of the heart, and to a change of structure in the substance of the heart itself. As for the treatment recommended in the various affections just mentioned, Dr. Reeder refers his readers to that pursued in cases of angina pectoris. The strictest diet, total absence from any other liquid than water, absolute quiet both of mind and body, venesection, diuretics, purgative medicines of the mildest kind. These are the means on which we should rely principally for relief. The foxglove and hydrocyanic acid will be found powerful auxiliaries in checking and keeping down the impetus of the circulation. The following case, related by Dr. Reeder, would seem to prove that considerable alteration in the organic structure of some parts of the apparatus of circulation may exist, as it were, in a latent state, and be brought into a formidable activity by any of those causes which tend greatly to increase the momentum of circulation.

"A man, of a robust habit of body, who was affected, on taking exercise, with violent palpitation of the heart, pain in the chest, and difficulty of breathing: his pulse was weak, quick, irregular, and intermittent; he was subject to epistaxis, and his face had a livid aspect; serous effusion into the chest and general anasarca took place, and, after the lapse of some time, he died. On dissection, the left auriculo-ventricular aperture was found greatly contracted, by reason of the formation of an osseous substance around its circumference, and which had produced dilatation of the left auricle and of the right ventricle and auricle, and effusion of water into the chest; the liver was also engorged with blood."

IV. *Enlargement of the Heart.*—This, Dr. Reeder observes, may arise from dilatation of its cavities, or from the addition of muscular substance, or from both conjointly. The experiments of Le Gallois, who measured the capacity of the right and left ventricle by the weight of a quantity of mercury employed to fill them, leave no doubt that the left cavity, or ventricle, is smaller than the right in all individuals. This cavity (the aortic,) is not unfrequently found dilated much beyond its natural dimensions, constituting what Corvisart has called "aneurism of the left ventricle." It is the most common of the organic affections of the heart, being found present in more than one-half of the individuals affected with those complaints.

An enlargement of the right ventricle is of rare occurrence; but not so with regard to the general enlargement of the heart, in which both the ventricles, as well as the auricles, are enlarged; though, even in this case, a certain proportion is observed between the enlargement of the right and left cavities, which corresponds to the differential proportion of their original or natural capacities.

The heart, however, may be enlarged from the addition of muscular and cellular substance, without at the same time having its cavities altered in their natural dimensions.

There is much obscurity yet in the investigation of the causes which may be supposed to produce all these enlargements. Baron Corvisart and Mr. Merat, one of his most distinguished pupils, have assigned several, and endeavoured to explain their *modus operandi.* Our author, who has, in most parts of his book, copied nearly verbatim those two writers, has no suggestion of his own on this important point.

We have met with an enlargement of the heart produced by a considerable deposition of caseous matter in the right ventricle, and by strong carneous bands stretching across both cavities, in a young lady, who died after an illness of thirty hours only, brought on by violent exercise; and whom we examined after death, in the presence of Dr. Doxat of Brussels. In this instance, it would have been in vain to have looked for any diagnostic symptom during life that could have led us to suspect the presence of so much disease.

Our readers have, before now, been reminded by us of Portal's Memoir on Dilatation and Aneurisms of the Heart. They will find in it, and in Corvisart's book, every fact brought forward by Dr. Reeder; particularly with regard to simple hypertrophy of the heart, or to dilatation of the ventricles, with increase of bulk in the texture of that organ.

As to the diagnosis of the various lesions we have enumerated, it would be a waste of time were we to attempt to analyze what Dr. Reeder has said on that subject, since our readers must have still fresh in their memory the brilliant, clear, and practical directions given by LAENNEC, of whose work on the Diagnosis of Diseases of the Heart, and on the use of the *Stethoscope* as a new means of exploration, we gave an extensive and minute account in our forty-third volume.

V. *Diminution in the Size of the Heart.*—"The heart has sometimes been found very small in comparison to the age and stature of the person, but the sanguiferous system, in some cases, of its proper magnitude; at least, not diminished in the same ratio with the size of the heart: while, in other instances, the diminution in these parts is proportional to each other.—This state of the heart, however, is rarely met with, and its existence cannot, with certainty, be detected during life, by any tokens with which we are at present acquainted. The body, however, will generally be rather small and delicate, and the pulse quick and weak."

This state of the heart has been chiefly found in those who have died of pulmonary consumption. Its presence cannot be ascertained during life; and no treatment, therefore, can be devised in order to change it.

VI. *Adhesion of the Pericardium to the Heart.*—Our author is very brief on this subject. Carditis, pericarditis, or rheumatism of the heart, will each produce adhesion of the pericardium to the heart. We were present at a dissection last year, in which the adhesion in question, the result of disease, was found to be as intimate and complete as that of the peritoneum investing the substance of the liver in a healthy subject.

VII. *Polypi.*—Dr. Reeder is one of those who do not admit the existence of cardiac polypi. He states that it is now generally believed that, in most instances, those concretions which have so commonly obtained the appellation of polypi, consist merely of unorganized coagulable lymph. We can only say, that we possess a most beautiful preparation of a regular polypus, found in the right ventricle of the heart, from whence it branched off into the right and left pulmonary arteries, through the ramifications of which it penetrated most distinctly. The centre of the polypus found in the ventricle seems to have been moulded in its outline by the parietes of the ventricle. It was of a reddish colour; but, by long maceration, has become perfectly white. It was subjected to the action of several known solvents, without experiencing the slightest alteration. The patient had died of what are commonly called puerperal convulsions, and was examined by myself, (in the presence of two other medical officers of the Westminster General Dispensary, where the preparation is kept,) with a view of ascertaining the cause, as well as the effect, of that formidable complaint; but, with the exception of the polypus in question, no sign of disease offered itself to our view in *any* part or *cavity* of the body.

We find, on looking over the scattered manuscript sheets that lie on the table before us, that we have suffered our desire of being impartial, and of giving our readers a full analysis of the contents of the book we analyze, to get the better of us; and we fear we have extended our account beyond the usual limits. We must therefore stop short, and be satisfied with referring our readers to the original for the contents of the concluding fifty pages of Dr. Reeder's book.

We cannot, at the same time, close our account of this work without expressing our surprise at two curious facts, connected with its performance, which have struck us from the first moment we sat ourselves down to the task of reviewing it. The first is, that the Preface, though signed with Dr. Reeder's initials, is written in a style and language so different from those of the work itself, and so inferior to them,—indeed so incorrect,—that we must conclude, either that the work is not composed by the person whose name appears in the title-page, or that the initials affixed to the Preface are not those of the author. The work itself is written in a plain, clear, and generally correct language: its construction is natural and grammatical; its sentences are aptly arranged, and the phrases almost always felicitous. But what can one say of the Preface, of which we extract the following passages at random?

"If we penetrate into that vast labyrinth of speculation in which the art of medicine had, for so many centuries, been deeply involved; or glance at that *proclivity* which had so long *invaded* the human mind, *to fabricate* hypotheses, as also to receive and adopt, with implicit credence, the theoretical doctrines of ingenious men; or survey that *rigid adherence*, or *bigotted deference*, which so many were formerly wont to *pay*, with the utmost sedulity, to the opinion and practice of their predecessors, thinking, no doubt, that they had attained the climax of all perfection;" &c. &c.

Here is Mrs. Malaprop with a vengeance! "To glance at a *proclivity* which invades the human mind;"—"the *adherence* which many are wont to *pay*!" &c. &c.

Farther on we have "the nature of diseases *substantialized*;" "a mind imbued with much *sapience*:" and, on the subject of the causes which retarded the progress of medical science, we find the following very intelligible, eloquent, and grammatical sentence:

"And there is little doubt *but what* such sophisms have, ever since the profession of medicine was cultivated as a science, been productive of the most baneful consequences, in having proved, to an immeasurable extent, a retardation to its improvement."

But what is really better than all these fragments, in our opinion, is the paragraph at page ix. which we give entire, because it will convince our readers, as it has convinced us, that the man who could scribble such trash could not compose so respectable a work as the one we have been analyzing.

"And further, when the disease is thoroughly understood, and is known to admit of a cure, it creates activity, promptitude, and perseverance in the practice of the medical attendant: while, on the contrary, when it is satisfactorily demonstrated to be of that nature wherein no recovery can be accomplished, it *emanates* a prescience to his mind *as to what* may be the termination of the case, in the place of which, perplexity, mingled with anxious conjecture, would in all probability reside, provided such knowledge had not been attained. This last-mentioned state, too, teaches the cultivator of our art, on some occasions, the humiliating lesson of remaining, as it were, a mere spectator, rather than becoming an officious interferer; or, at least, if any thing should be attempted where these physical impossibilities exist, it will only be with the view of palliating the patient's sufferings, and smoothing his path to that destination *whereat* he must ere long finish his terrestrial career."

The second fact to which we allude is the entire omission of Laennec's name, or of any reference to his work, as if the book now before us had been written previously to the appearance of Laennec's Treatise, by some one who, before he could profit of the French physician's publication, had perhaps proceeded "to that destination *whereat*" we all must finish our terrestrial career.

---

*A Treatise on the Art of Cupping: in which the History of that Operation is traced; the Complaints in which it is useful indicated; and the most approved Method of performing it described.* By Thomas Mapleson, Cupper to his Majesty. Second Edition, considerably improved. 12mo. pp. 94. Callow, London. 1821.

If books were to be judged by their size, that which is now lying before us could scarcely be deemed deserving of consideration. A thin *libellus*, craving for admission into the vast repositories of thick octavos and ponderous quartos belonging to the faculty, in the humble garb of a duodecimo, would stand but a poor chance of a courteous reception, did we not know that much valuable information may be concentrated in a few words; and that a man who is really master of his subject, can impart it to others without having recourse to hot-pressed vellum, wide margins, and an interminable string of chapters, which can only be understood by the assistance of a copious analytical table.

The author of the small pamphlet we are about to review, is well known to the medical practitioners of the metropolis. There is scarcely a physician, surgeon, or apothecary of note, practising among the respectable classes of society, but must, at some time or other, have claimed the assistance of Mr. Mapleson, and seen the surprising dexterity with which that assistance has been afforded. With Mr. Mapleson, the operation of cupping is divested of all its objections, is free from pain, and the reverse of tedious. We have seen him perform it a hundred times, and each time with equal ability and success. He has positively no rivals in this humble branch of surgery, though we are aware that there are many other cuppers of most respectable qualifications.

When it is considered that, in the majority of acute and life-threatening diseases, the abstraction of blood by cupping has been found of the highest importance and advantage,—that at all ages, and in both sexes, the operation may be performed with the certainty of success,—and that, in the case of some of the most formidable disorders of children, cupping has saved them from destruction,—it will be admitted that Mr. Mapleson is correct in wishing to disseminate, by means of the press, the necessary instructions for the able performance of that operation; and that we are not to be accused of trifling with our readers, for noticing a performance which might otherwise have escaped their consideration.

This little book is divided into three parts. The first contains an Historical Sketch of the Operation of Cupping; the second, a Synopsis of the Diseases in the Treatment of which Cupping has been found useful; and the third, Practical Instructions for performing the Operation.

1. *History of the Art of Cupping.*—The rudest attempt made in early ages to practise this branch of surgery, seems that which is noticed by Hippocrates, and which consisted in applying, over the scarified part, a small gourd or cucurbit, furnished with two orifices: one of sufficient size to comprehend the scarifications; the other very small, for the purpose of exhausting the air by suction. There was also another contrivance for exhausting the air of a brass cup placed over the scarified part,—namely, by means of a piece of burning flax, linen, or tow, introduced into the cup; which method corresponds to that employed for making a vacuum in the cupping-glass at the present day. If we are to believe Herodotus, the practice of abstracting blood by means of scarifications, quickened by the application of exhausted glasses, seems to have been prevalent among the Egyptians several hundred years before Christ, and long before the time of Hippocrates. Not unlike many modern Sangradoes, the Egyptians considered the abstraction of blood as a remedy for almost every species of disease. Among the Romans, the utility of cupping appears to have been properly appreciated, and the practice of it to have been general. Celsus has given us a correct and elegant description, both of the instrument employed, and of the operation, in his chapter entitled "De Sanguinis Detractione per Cucurbitulas." This fact has not escaped the notice of Mr. Mapleson, who has quoted the passages in question, with becoming commendation. Our author has even dipped into the "Arabian Nights" for historical information respecting his favourite art, and found that the chattering barber possessed, among other qualifications, the art of cupping.

This operation, indeed, as a remedy for disease, appears to have spread all over the world. In India, it is performed by natives, generally females, who continue to use the same means as the Egyptian cuppers. The late Mungo Parke, in his travels into the interior of Africa, saw cupping practised with a bullock's horn. In the islands of the South Sea, and in New Holland, local extraction of blood is prevalent; and consists in applying the mouth to scarifications previously made by incisions with a sharp reed. From the account given by Lionel Wafer, it appears that the American Indians were in possession of the art of abstracting blood by superficial scarifications, which were made by little arrows shot against various parts of the naked body of the patient.. Negroes, newly imported from Africa into the West Indies, have been known to make punctures, from which they would suck the blood by the intervention of a gourd, with a view to counteract the bad effects of bruises, We have the testimony of Galen to prove that cupping and scarifying was practised during the dark ages.

Mr. Mapleson says, that one of the most important improvements in his art is the invention of the spring-box, by means of which a number of incisions are made at once, instead of being done in succession by the lancet or razor. Mr. Mapleson does not know of the existence of any account of this invention at an earlier period than that mentioned in Heister, the first edition of whose works was published about the year 1710; and he has himself had spring scarificators in his possession, the workmanship of which clearly indicated them to have been made about that period.

"Until little more than a century ago, scarification and cupping appear to have been operations performed by the regular surgeon, when deemed necessary. About that period the use of warm baths was introduced into this country by a person who had resided some years in Asia, and which still continue to be designated nearly by their original appellation, *haumaum*, the Turkish appellation for a warm bath, corrupted in common parlance into hummums. As these baths were copied from those of Egypt, a country to which I have endeavoured to trace the origin of scarification and cupping, which were there generally performed in the warm bath, so, when the practice of warm bathing was introduced into this country, the practice of cupping accompanied it."

Mr. Mapleson quotes, both from the Tatler and Spectator, some curious observations, to show the manner in which bathing and cupping were first made public in England.

"The Queen's Bagnio,* in Long Acre, is made very convenient for both sexes, to sweat and bathe privately every day, and to be cupped in the best perfection, there being the best and newest instruments for that purpose. Price 5s. for one single person; but, if two or more come together, 4s. each.—There is no entertainment for women after twelve o'clock at night; but all gentlemen who desire beds may have them at 2s. per night.

"Persons may be cupped at their own houses. The way of cupping is the very same as was used by the late Mr. Verdier, deceased.

"Wash-balls, perfumed, camphired, and plain, shall restore complexions to that degree, that a country fox-hunter, who uses them, shall, in a week's time, look with a courtly and affable paleness, without using the bagnio or cupping.

"Air-pumps,† single and double barrelled, with apparatus for demonstrating the several properties of the air.—Small air-pumps, with glasses for the new way of cupping; scarificators, one of which makes at once ten, another thirteen, another sixteen, effectual incisions."

The operation having fallen into contempt by thus getting into the hands of low mercenary fellows, both physicians and surgeons neglected it: the former, because patients had recourse to it without previous advice; and the latter, because, being performed by others, their professional profits were necessarily diminished.

"Of late years, however, the utility of this local abstraction of blood has been recognized by all the more enlightened and eminent practitioners of both medicine and surgery. The practice has, consequently, been rescued from that class of inferior practitioners into whose hands it had fallen. Men of experience have devoted themselves to this peculiar operation; among whom I trust I may be allowed to class myself as an humble individual, an instrument, I hope, for good, in the hands of Providence. Still the adroit performance of this operation, simple as it may appear, continues to be confined to a few individuals in the metropolis. My purpose, in committing these few pages to the press, is to render more extensive the practice of an operation, now generally acknowledged to be in many cases essentially useful, and which the remarks contained in the subsequent pages, especially if aided by a very few practical lessons, will, I trust,

---

* *Tatler*, vol. ii. No. 95, p. 429, Chalmers's edit.
† *Spectator*, vol. iv. No. 289, note.

enable any man, possessed of common ingenuity, to perform with propriety, and even elegance."

II. *Diseases in which Cupping has been employed.*—It does not follow, because cupping has been ordered by various practitioners in all the diseases which form the long list given by our author, that the benefit resulting from that operation in those diseases must be undoubted. Mr. Mapleson says, that he has found cupping very useful in particular kinds of head-ache; and we have seen particular kinds of head-ache made worse by cupping. To enumerate, therefore,—and still more so, to specify by their individual names,—the complaints in which the operation of cupping is likely to be of service, is to advance more, in favour of that operation, than experience warrants us in adopting. Besides, it could not be expected of our author that he should even enter on this part of his present performance; for, as in most cases, he is deprived of the means of knowing the ultimate result of the disease in which he has been directed to apply the cupping-glasses, his report respecting the success of cupping in *any* disease cannot be received as any thing of much authority. We regret, therefore, to find such a report in this book, and, what is still worse, we are sorry to see it drawn up in so positive a language; for it will, we fear, produce mischief both among those patients who, from sordid motives, would rather save the paltry fee of a medical attendant; and among the inferior, yet numerous, class of practitioners, who may be disposed to take Mr. Mapleson's book for their guide as to the propriety of having recourse, or not, to the operation of cupping.

Our readers will see the justice of our remarks when they shall have cast their eyes on the long catalogue of diseases in which cupping is stated by Mr. Mapleson to have been generally employed with advantage:—"Apoplexy; angina pectoris; asthma; spitting of blood; bruises; cough; catarrh; consumption; contusion; convulsions; cramp; diseases of the hip and knee joints; deafness; delirium; dropsy; epilepsy; erysipelas; eruptions; frightful dreams; giddiness; gout; hooping-cough; hydrocephalus; head-ache; inflammation of the bladder, bowels, eyes, liver, and lungs; intoxication; lethargy; loss of memory; low spirits; lumbago; lunacy; measles; nervous complaints; numbness of the limbs; obstructions; ophthalmia; pleurisy; palsy; defective perspiration; peripneumony; rheumatism; to procure rest; sciatica; shortness of breath; sore throat; pains of the side and chest."

It is, however, just to observe, that Mr. Mapleson disclaims any intention of intruding on the province of the regular practitioner of medicine, by pretending to direct in what complaints cupping should be used. Indeed, our preceding observations are intended less as a reproach to the author, than as a caution to his readers not to be misled by the apparent simplicity with which disease and remedy have been made to go hand in hand in the book under our present consideration.

Still, even in this part of Mr. Mapleson's little treatise, there are a certain number of facts which, coming from a man of so much experience and well-known integrity, ought to have some weight with the faculty.

In obstinate ophthalmia, Mr. Mapleson has found cupping preferable to the application of leeches.

"Indeed, several cases have occurred to me, where the redness of the eyes seemed to be augmented, and the sense of fullness to the feelings of the patient increased, after the application of leeches to the temples; when, by applying a cupping-glass over the part bitten by the leech, and thus taking away more blood, immediate relief has been produced."

The swelling of the eye-lids, produced sometimes by the application of leeches, is completely obviated by applying a cupping-glass immediately after the removal of the leech.

In suppression of catamenia, the application of cupping-glasses to any part of the abdominal extremities is said to be eminently successful. Mr. Mapleson knows, we dare say, how painful the operation is when practised on the gastrocnemii muscles.

In phrenitis, cupping is the most energetic means of depletion. One hundred ounces of blood have thus been removed, and the issue is said to have been successful.

"In apoplexy, when the patient is comatose, and cannot readily change his posture, I prefer taking blood from the temples; and have frequently drawn twenty, and even thirty, ounces, without materially lowering the pulse. Two cases of this description occurred to me very lately, where, under the direction of an eminent surgeon, the patients, who were females rather advanced in life, were each of them cupped to the extent of one hundred ounces of blood within a week."

In hydrocephalus, (we prefer calling it cephalitis, for, when cupping is of service, effusion of serum has not yet taken place,) Mr. Mapleson has performed the operation of cupping hundred of times.

But, of the various effects of cupping, the most extraordinary which Mr. M. has witnessed, is the effect of that operation, when performed in the vicinity of the head, in almost immediately suspending the state of intoxication in consequence of taking too large a quantity of fermented liquor.

"To illustrate this subject, I shall, from many cases within my knowledge, detail the following circumstances, which occurred some months ago. By the desire of a physician, I repaired, about ten o'clock at night, to a celebrated tavern, where we found four gentlemen, one of whom was laid on a sofa; his face extremely red, his eyes suffused with tears, the pupils dilated, and his knees every half-minute drawn up to his chin by violent spasmodic convulsions. Although the state of their companion was such as to create alarm, not one of the party could articulate sufficiently plain to give any account of what had occasioned it; but we learned from the waiter that a great deal of wine had been drunk.

"I was desired to take blood freely from the shoulders: in a short time after the operation was over, the gentleman, who was the youngest of the company, perfectly recovered his senses, and stated every circumstance that had occurred; that they had been hunting all the morning, and had hastily taken a good deal of wine upon an empty stomach, a condition in which it is very apt to induce sudden intoxication. He soon became so decidedly sober as to be able to see his companions safely home in a coach."

Like a skilful advocate, who leaves the strongest of his arguments for the peroration, Mr. Mapleson concludes his eulogium on cupping by the following striking and encouraging example:—

"A gentleman, who is now in his 101st year, calls at my house generally twice or oftener in the year. He loses ten ounces of blood, and walks home, a distance of three miles, without inconvenience. He has enjoyed good health for the greater part of his life, and has no complaint at present but occasional vertigo."

III. *Operation of Cupping.*—Will it be believed, after all that has been said of this operation, that the correct performance of it is at present confined within narrow limits; and, according to our author, can hardly be said to extend beyond the boundaries of the metropolis of these realms. We know not how matters are ordained in regard to this operation in the medical department of the army; but we can assure Mr. Mapleson and the public, that the medical officers of the royal navy, who have incontestibly given the *elan* to military surgery in this country, (for their toils and their triumphs long preceded those of the land-service,) have, to our knowledge, been in the habit of performing, for many years, the operation of cupping on their own patients; for which purpose they supply themselves with the necessary apparatus, agreeably to the rules of the service.

In Scotland and Ireland, the art of cupping is not practised as a distinct profession; and our author has been informed that it is rarely, if at all, recommended even in Edinburgh, that *supposed* centre of medical information.

In Paris, the operation is hardly known. The cupping apparatus is not to be met with. We have seen Baron Larrey scarify an inflamed surface, by drawing very nimbly over it, in various directions, a keen-edged scalpel, held lightly between the fore-finger and thumb, and apply over the part glasses which had been previously exhausted by burning a small quantity of tow within them. Of late years, however, a Dr. Salandriere invented a small apparatus, consisting of a moveable axis, armed at one extremity with one or more lancets, within an oblong glass, to one side of which an aspiring pump is appended; which apparatus is intended to supply the application of leeches.

Mr. Mapleson prefaces his instructions for the performance of cupping with a *rationale* on which the operation depends, and which he explains by the familiar examples of atmospherical pressure in the pump and barometer. If a cup, the air of which has been exhausted by the momentary introduction of the flame of a spirit-lamp, be applied to a given part of the surface of the body, the skin will rise up in it, by the pressure of the air upon the general surface of the body. Under this circumstance, the cutaneous and superficial blood-vessels become distended; a larger quantity of blood rushes into them; and, when the cup is re-applied after scarification, a much larger quantity of blood is discharged than could have been obtained without the vacuum.

We shall now quote, verbatim, the instructions for performing the operation with adroitness and success, as we find them in Mr. Mapleson's little book; trusting that the wide circulation of our Journal will materially assist in promoting the great object which the author professes to have had in view when he published his excellent treatise.

1. "The first step in the operation of cupping is to produce a partial vacuum over one or more portions of the surface of the skin.

2. "The mode now, I believe, universally adopted by regular cuppers, is the momentary introduction of the flame of a spirit-lamp, with a thick wick: the larger the glass, (if properly exhausted,) the less pain does the patient suffer, and the more freely does the blood flow.

3. "When about to perform the operation, let there be provided a hand-basin with warm water, a piece of fine sponge, and a lighted candle. Place as many glasses in the basin as may be judged requisite to obtain the quantity of blood intended to be taken away. If sixteen or twenty ounces are ordered, four glasses, of a size adapted to the surface, will in most cases be required. Each glass is then separately to be held, for an *instant*, over the flame of the spirit-lamp, and immediately placed upon the skin of the patient. Upon the quickness with which this is effected, depends the whole neatness and efficacy of the operation.—To obviate their want of dexterity, many operators in the country throw a little bit of tow or paper, dipped in spirits and inflamed, into the cupping-glass, the moment before it is applied; a very clumsy expedient, often adding unnecessarily to the sufferings of the patient by cauterizing the skin; doing harm, also, by rarefying the air more than necessary within the glass, in consequence of which the edges of the cup compress the cutaneous vessels so much as to obstruct the influx of the blood.

4. "If the glasses have been duly exhausted, the skin will be seen gradually to swell up within the cup, owing to the pressure of the air upon the parts in the vicinity, as well as to the expansion of the fluids contained in the cellular membrane. The skin becomes also of a dark purple colour, owing to the influx of blood into the smaller vessels. If dry cupping be only intended, the glasses may be allowed to remain on the skin for a few moments, and replaced five or six times, varying their position a little, to prevent bruising the skin. If the intention be to scarify and take away blood, the glass ought not to remain more than a minute; when it is to be removed by gently introducing the nail of the fore-finger under the edge, and the scarificator *instantly* applied, and the lancets discharged upon the skin, before the tumor has had time to subside. Upon the rapidity or slowness with which the application of the scarificator succeeds the removal of the glass, depends all the sufferings of the patient. If the skin has completely subsided before the stroke of the lancets, much unnecessary pain is inflicted.

5. "The glasses are thus to be removed and re-applied successively. They should be a second time removed, if necessary, as soon as the blood is perceived to coagulate within them, or when they are so full as to be in danger of dropping off. For the sake of neatness, care should be taken to insert the nail under the upper part of the glass, and open them downwards; gently wiping the wounds at the same time with a warm moist sponge.

6. "The glasses, previous to every application, should be rinsed in the warm water, but not dried. To obviate the unpleasant sensation produced by the coldness of the metal, it is advisable to pass the instrument for a moment over the flame of the lamp before using.

7. "When the operation is finished, it is common to apply a piece of fine linen rag to the wounds; but, if the patient does not object to a little smarting, either arquebusade water or spirits of wine is a preferable application, as it immediately stops the oozing of the blood, promotes the healing of the wounds, and prevents the subsequent itching, which I have heard some patients complain of, as the most unpleasant part of the operation.

8. "It is a common error to make the incisions too deep, especially if the object be to take away much blood; being convinced that nothing is gained by going deeper than the cutis, or true skin, while an unnecessary increase of pain is caused to the patient.

9. "It is certainly preferable to make the incisions in the direction of the fibres of the subjacent muscles; but it is not of much importance, as, in my opinion, the incisions ought never to penetrate so deep as the muscular flesh.

10. "No step in the operation of cupping demands more the attention of the operator than the state of his scarificators. If not exquisitely keen, they occasion unnecessary pain; if foul or rusty, they may communicate disease, or give rise to festering sores. The lancets or cutters should be kept as sharp and in as fine order as possible, so as to make a clean incision, without bruising or giving fruitless pain.

11. "The intention of the operation is, I believe, frequently defeated, from want of attention to these apparently trivial circumstances."

---

### CRITICAL ANALYSIS

OF

